# Structural synaptic signatures of Alzheimer's disease and dementia with Lewy bodies in the male brain

**DOI:** 10.1111/nan.12852

**Published:** 2022-10-09

**Authors:** Oleg O. Glebov, David Williamson, Dylan M. Owen, Tibor Hortobágyi, Claire Troakes, Dag Aarsland

**Affiliations:** ^1^ Institute of Neuroregeneration and Neurorehabilitation Qingdao University Qingdao Shandong China; ^2^ Department of Old Age Psychiatry, Institute of Psychiatry, Psychology and Neuroscience King's College London London UK; ^3^ Randall Centre for Cell and Molecular Biophysics, Faculty of Life Sciences and Medicine King's College London London UK; ^4^ Institute of Immunology and Immunotherapy, School of Mathematics and Centre of Membrane Proteins and Receptors (COMPARE) University of Birmingham Birmingham UK; ^5^ ELKH‐DE Cerebrovascular and Neurodegenerative Research Group and Department of Neurology, Faculty of Medicine University of Debrecen Debrecen Hungary; ^6^ London Neurodegenerative Diseases Brain Bank, Department of Basic and Clinical Neuroscience, Institute of Psychiatry, Psychology and Neuroscience King's College London London UK; ^7^ Centre for Age‐Related Medicine (SESAM) Stavanger University Hospital Stavanger Norway

**Keywords:** Alzheimer's disease, dementia, Lewy body dementia, neurodegeneration, synapse

**Key Points**

- Confocal and super‐resolution microscopy reveals alterations in synaptic organisation associated with neurodegeneration in *post mortem* human brain samples.
- Different types of dementia are associated with distinct changes in synaptic structure.
- The observed changes are restricted to male brain samples.

Alzheimer's disease (AD) and dementia with Lewy bodies (DLB) are the two major forms of neurodegenerative dementia, characterised by distinct pathological hallmarks involving aggregation of misfolded proteins. One proposed key pathophysiological mechanism in AD and DLB, as well as in other neurodegenerative disorders, is synaptic dysfunction.^1^, ^2^, ^3^, ^4^, ^5^, ^6^, ^7^, ^8^ The details of human synaptic pathology in AD and DLB besides synaptic loss are, however, poorly characterised, and the underlying processes remain unknown.

Our previous studies identified distinct patterns of aberrant protein expression in AD and DLB,^5^, ^9^ raising the possibility that manifestation of AD and DLB may differ at the local synaptic level. To directly address this, we employed immunostaining, confocal and super‐resolution microscopy to visualise synaptic structure in *post mortem* human brain isolates (Figure S1). Samples represented Brodmann Area 9 (BA9), a key part of the prefrontal cortex implicated in neurodegeneration,^5^ from a cohort of 32 control subjects and cases with severe AD and DLB.

We first assessed synaptic structure in 24 cases (Table S1.1, Figure S2) by immunostaining for presynaptic active zone (AZ) protein Bassoon (Bsn) and postsynaptic density (PSD) protein Homer. The Homer/Bsn ratio showed no correlation with the *post mortem* interval (PMI), suggesting that synaptic structure was largely unaffected by preparation and storage (Figure S2c). There were no significant differences in Bsn levels in Bsn‐positive puncta, in area of Bsn‐positive puncta, in levels of Homer or in levels of another AZ protein Rab‐interacting molecule (RIM) (Figures 1A and S3a–c,e), indicating that AD or DLB did not majorly alter the general morphology of the AZ and PSD.

**FIGURE 1:**
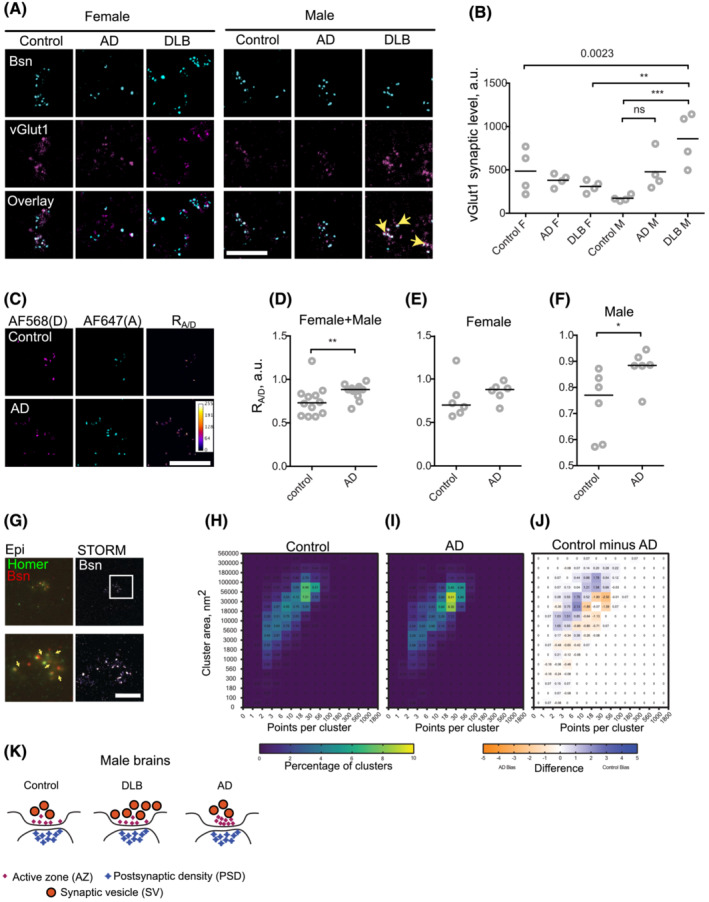
Disease‐specific structural signatures of dementia in the male brain. (A) Representative confocal microscopy images of human brain synaptoneurosomes fixed and immunostained for Bassoon (Bsn) (cyan) and vGlut1 (magenta). Scale bar, 10 μm. (B) vGlut1 synaptic levels in control, Alzheimer's disease (AD) and dementia with Lewy bodies (DLB) samples from female (F) and male (M) brains. ***p < 0.0001, **p < 0.01, one‐way ANOVA and Holm–Šidák's post‐test. (C) Representative images of synaptoneurosome samples labelled for a ratiometric Bsn clustering assay. Scale bar, 10 μm. (D) Ratiometric clustering measured in control vs AD samples. **p < 0.0083, Mann–Whitney's test. (E) Ratiometric clustering in samples from female brains shows no significant difference between control and AD. *p* = 0.4399, *t*‐test. (F) Ratiometric clustering in male brains shows significant difference between control and AD. *p < 0.05, *t*‐test. (G) Representative image of the synaptoneurosome preparation immunostained for Homer (Alexa Fluor 488, green) and Bsn (Alexa Fluor 647, red). Arrows show multiple instances of Bsn puncta overlaying or adjacent to Homer puncta, indicative of synaptic structures. The right panels show the corresponding STORM image of the Bsn channel; scale bar, 5 μm. (H) Area and size of Bsn clusters in control samples, expressed as a normalised 2D histogram heatmap for the control condition. (I) Same for AD samples. (J) The difference in relative distribution of clusters into the histogram bins between the control and AD conditions. *N* = 3036 and 4153 clusters for control and AD, respectively, 4 brains per condition. (K) A putative model of disease‐specific presynaptic remodelling in male Brodmann Area 9 (BA9) synapses with AD and DLB. DLB results in increased recruitment of vGlut1. AD results in an increased density of active zone (AZ) matrix clustering, whereas postsynaptic density (PSD) remains relatively unaffected by either DLB or AD.

We then performed double immunostaining for Bsn and vGlut1, a key component of synaptic vesicles (SV) that has been implicated in AD, as well as in Parkinson's disease (PD), which shares many neuropathological features of DLB. Synaptic vGlut1 levels were elevated in both AD and DLB male samples compared with the controls, but the increase in AD samples was not statistically significant. In contrast, synaptic vGlut1 levels were significantly increased in male DLB samples compared with male controls or female DLB samples (Figures 1A,B and S3d). Therefore, we conclude that vGlut1 is specifically enriched in synapses from male subjects with DLB.

Recent evidence shows that neuronal activity regulates nanoscale clustering of the AZ^10^; we therefore reasoned that aberrant synaptic activity in AD may lead to nanoscale changes in AZ architecture and investigated this in an extended cohort including 24 control and AD female and male cases (Table S1.2, Figure S4). We assessed Bsn clustering using confocal microscopy and a ratiometric assay developed by us previously^10^ (Figure S5a). Clustering in AD samples was significantly increased in male but not in female brains (Figure 1C–F), indicative of an increased density of Bsn packing within the AZ. Clustering did not significantly correlate with age (Figure S4d), suggesting that the difference between control and AD samples was not due to the difference in median age (Figure S4a).

Finally, we used super‐resolution imaging and machine learning clustering analysis developed by us^11^ to directly quantify Bassoon clustering in four control and four AD male brains. A decrease in median cluster area and an increase in the median number of clusters were observed (Figure 1G–J). However, nested two‐tailed *t*‐tests showed no significant difference between median values in control and AD samples (Figure S5b,c), indicative of considerable variability in super‐resolution data obtained from *post mortem* human brain samples. Taken together, these data show that AD in the male brain is associated with nanoscale reorganisation of presynaptic architecture.

Our findings show that two major forms of dementia are associated with distinct changes in presynaptic organisation, providing direct evidence for disease‐specific synaptic defects in neurodegeneration (Figure 1K); furthermore, we present evidence for sex‐specific effects in dementia at the level of synaptic structure, in line with association between biological sex and clinical manifestation of dementia.^12^, ^13^ Some data from female samples showed similar trends to those observed in male samples, yet failed to reach the threshold of significance (Figure 1E,F), possibly reflecting the higher incidence of ‘pure’ dementia in men vs a mixed LBD/AD pathology reported in women.^14^ It remains to be determined whether the observed changes represent a direct effect of pathology or a form of compensatory synaptic plasticity.^3^

In order to enhance the exploratory power of the approach described in this paper, further investigation will require larger case cohorts combined with testing for multiple synaptic markers, with particular relevance for quantitative analysis of nanoscale synaptic structure. Thus, our initial observations reported here pave the way for deeper investigation of synaptic architecture in neurodegeneration, with the long‐term potential for development of targeted diagnostics and therapies for dementia.^3^ Last but not least, in‐depth structural analysis of human synaptic dysregulation will allow for knowledge‐based validation of animal models,^15^ providing a much‐needed boost for experimental investigation and therapeutic development in neurodegeneration.

## CONFLICTS OF INTEREST

None declared.

## ETHICS STATEMENT

Freshly frozen brain samples were provided by the London Neurodegenerative Diseases Brain Bank (KCL), part of the UK Brain Banks Network. The project was carried out under the ethical approval of the tissue bank (18/WA/0206 REC for Wales). Written informed consent from the donors and/or their relatives as appropriate was obtained by the London Neurodegenerative Diseases Brain Bank.

## AUTHOR CONTRIBUTIONS

The project was conceived by O.O.G., T.H. and D.A. Experimental methodology was developed by O.O.G., D.W. and D.M.O. Experimental procedures were carried out by O.O.G. and D.W. Experimental data analysis and interpretation were performed by O.O.G., D.W. and D.M.O. Clinical data were analysed by O.O.G., T.H., C.T. and D.A. The manuscript was drafted by O.O.G. and written with contributions from all authors.

### PEER REVIEW

The peer review history for this article is available at https://publons.com/publon/10.1111/nan.12852.

## Supporting information


**Table S1.** Summary of clinical cases.
**Table S2.** Primary antibodies used in the study.

**Figure S1.** **Characterization of the synaptic preparation**. **a**, Schematics of the preparation of neurosynaptosomes. **b**, Colocalization between synaptic markers in neurosynaptosomes. **c**, Median synaptic area in neurosynaptosomal fraction (Fraction) and non‐homogenised samples (Whole) from 4 brains. P = 0.3881, t‐test. **d**, Synaptic Bsn labelling in neurosynaptosomal fraction and non‐homogenised samples from 4 brains. Intensities were normalised to neurosynaptosomal fraction. P = 0.5036, one sample t‐test. **e**, Synaptic vGlut1 labelling in neurosynaptosomal and non‐homogenised samples from 4 brains. Intensities were normalised to neurosynaptosomal fraction. **P = 0.0012, one sample t‐test.

**Figure S2.** **Supporting data for Table 1.1**. **a**, Age is not significantly different between groups. P = 0.7263 (male), P = 0.5430 (female), P = 0.7097 (both), 1‐way ANOVA. **b**, PMI is not significantly different between groups P = 0.8849 (male), 0.6154 (female), 0.7826 (both), 1‐way ANOVA. **c**, Postsynaptic/presynaptic ratio does not correlate with PMI. P = 0.4341, r = −0.1675, Spearman's correlation coefficient.

**Figure S3.** **Supporting data for synaptic marker proteins levels 1. a**, Bsn synaptic levels in control, AD and DLB samples from male and female brains P = 0.5210, 1‐way ANOVA. **b**, median synaptic area in samples from control, AD and DLB male and female brains P = 0.1005, 1‐way ANOVA. **c**, RIM synaptic levels in sample from control, AD and DLB female and male brains P = 0.3139, 1‐way ANOVA. **d**, vGlut1 synaptic levels in samples from control, AD and DLB female and male brains, second sample preparation. **P < 0.01, *P < 0.05, 1‐way ANOVA and Holm‐Šidák's post‐test. **e**, Homer synaptic levels in sample from control, AD and DLB female and male brains. P = 0.7449, 1‐way ANOVA.

**Figure S4.** **Supporting data for Table 1.2**. **a**, Age of AD cases was significantly higher than that of control cases. *P < 0.05, Student's t test. **b**, 1‐way ANOVA divided sex and condition shows no significant differences. P = 0.1176, 1‐way ANOVA. **c**, PMI is not significantly different. P = 0.6761, t‐test. **d**, R_A/D_ does not correlate with age of cases. P = 0.1374 (Control), P = 0.7429 (AD), r = −0.4526 (Control), r = 0.1058 (AD), Spearman's correlation coefficient.

**Figure S5.** **Supporting data for clustering experiments**. **a**, Schematics of the ratiometric clustering assay – adapted from Ref. 8. **b**, Area of Bsn clusters in control and AD samples; pairwise comparison is a two‐tailed nested t test. **c**, Localization counts for Bsn in control and AD samples; pairwise comparison is a two‐tailed nested t test.

## Data Availability

For the materials and methods, see the supporting information. The datasets generated and analysed during the current study are available from the corresponding author on reasonable request.
