# Supplementary material for: Structural synaptic signatures of Alzheimer's disease and dementia with Lewy bodies in the male brain
Source: Neuropathol Appl Neurobiol. 2022 Oct 9;49(1):e12852. doi: 10.1111/nan.12852 (PMC10092423; doi:10.1111/nan.12852)
Supplement: Supplementary file 1 — Table S1. Summary of clinical cases. Table S2. Primary antibodies used in the study. [file NAN-49-0-s004.docx]

# Supplementary information

## Materials and Methods

### Post mortem human brain samples

Eight AD, eight DLB and eight control cases were included, with further four control and four AD cases included for extended analysis of synaptic structure in AD. Selection and diagnostic procedures have been described elsewhere. All cases were diagnosed clinically with AD or DLB and this was confirmed by detailed neuropathological examination. All AD cases were classified as severe (Braak stage IV-VI), and all DLB cases exhibited diffuse neocortical pathology. Control cases showed no clinical evidence of cognitive decline and demonstrated only mild age-related pathology (Braak stage no more than II) on neuropathological examination.

Further details for all cases are presented in **Table 1**.

### Biochemical preparation of the brain tissue

Tissue samples were dissected from the grey matter but may have contained small amounts of white matter. Small quantities (20-50 mg) of tissue from the cortex (Brodmann Area 9) were excised using a scalpel and homogenised in a Dounce homogeniser for 20 strokes on ice in 300 µl of homogenisation buffer, consisting of phosphate buffered saline (PBS), phosphatase inhibitor cocktail, protease inhibitor cocktail, and 5 µM EDTA). The resulting suspension was transferred into 1.5 ml Eppendorf tubes and the large cell debris sediment was pelleted at 1000g at the benchtop microcentrifuge for 10 min at 4°C. The supernatants containing brain synaptoneurosomes were diluted with the homogenisation buffer to the same concentration (corresponding to 0.05 mg of the original brain material per 1 µl of buffer), aliquoted into 20 µl aliquots and stored at -70°C.

### Immunocytochemistry

Synaptoneurosome extracts (20 µl) were added to 35 mm Petri dishes containing 1.5 ml of 2% paraformaldehyde (PFA) in PBS and 2-3 12 mm glass coverslips (thickness 1.5) coated with poly-L-lysine; to pellet the fixed material, Petri dishes were then centrifuged in the cell culture benchtop centrifuge for 20 min at 2800g. Coverslips were washed 4 times in excess of PBS and permeabilized in 0.2% Triton-X100 in PBS supplemented with 5% horse serum for 10 min. For immunostaining, coverslips were transferred into individual wells of a 24-well plate. Subsequent incubations were carried out in the permeabilization buffer. Coverslips were incubated with appropriate primary antibodies (see Table 2) for 60 min at room temperature (RT), washed 4 times in PBS and incubated with Alexa Fluor 488 and Alexa Fluor 647 conjugated secondary antibodies as appropriate at a concentration of 0.2 μg/mL each for 60 min at RT. Coverslips were then washed 4 times in PBS, mounted in Fluoromount-G mounting medium (Southern) on microscopy slides and imaged on a Zeiss LSM710 microscope equipped with a standard set of lasers through a Plan-Apochromat 63x/1.4 Oil objective. The imaging system was controlled by ZEN software. Regions of interest sized 1024 by 1024 pixels (65.8 nm/pixel) were imaged at speed 7 with the averaging setting 2. Pinhole size was kept to 1-2 Airy units. Excitation laser wavelengths were 543 and 633nm. Bandpass filters were set at 570-650 nm (Alexa Fluor 488) and 650−750nm (Alexa Fluor 647). Image acquisition was carried out at the 12-bit rate. Settings were optimised to ensure appropriate dynamic range, low background and sufficient signal/noise ratio.

### Ratiometric clustering assay

Coverslips were processed as described above for immunocytochemistry and incubated with a 1:1 mixture of donor- and acceptor- conjugated secondary antibodies at a concentration of 0.2 µg/ml each for 60min at RT. Alexa Fluor 568 and Alexa Fluor 647 were used as donor and acceptor respectively. Coverslips were then mounted in mounting medium (Southern), allowed to dry for 30min at RT and imaged on a Zeiss LSM710 microscope equipped with a standard set of lasers through a 63x oil objective. Excitation wavelengths were 543 and 633 nm. Bandpass filters were set at 560-615 (Alexa Fluor 568) and 650-750 nm (Alexa Fluor 647). Image acquisition was carried out as described above.

### Confocal microscopy and image analysis

To identify individual synapses, images were thresholded in ImageJ using the “Moments” setting, resulting in binary images, and particles were then counted automatically using the “Analyze Particles” command across the whole image. Visible areas of non-specific fluorescence associated with tissue debris were excluded from analysis. Thresholded data from the Bassoon (Bsn) channel was used to determine synaptic locations as Regions Of Interest (ROI). Signal intensities were quantified for each synaptic puncta using the ROI Manager function. To avoid rare overlap of multiple synapses, only ROIs with areas ranging from 0.1 to 2 μm^2^ were included in further analysis. All values of circularity were included in analysis. Mean intensities were then calculated for each synapse, and the median value describing the distribution of synaptic intensities in the whole sample was reported for further statistical analysis. Background subtraction was performed as required. To quantify synapse-specific ratios R_A/D_, puncta of fluorescence were individually processed in the same manner.

### Validation of the synaptoneurosomal preparation

Immunohistochemical analysis of synapses in intact brain structure is associated with considerable artefacts due to incomplete reagent penetration and signal density, complicating both conventional and super-resolution microscopy^16^. Therefore, we opted for biochemical purification of the synaptoneurosome fraction (**Figure S1a**), which isolates structurally intact synapses and has been well established in structural and functional studies of brain synapses^17–20^. To visualise synapses, we used immunostaining for Bsn (see main text).

Bsn-positive puncta colocalised with the key classes of synaptic markers; these included inhibitory postsynaptic density (PSD) protein Gephyrin, excitatory postsynaptic density protein Homer, presynaptic vesicle (SV) glutamate transporter vGlut1 and voltage-gated calcium channel (VGCC) Cav2.1 (**Figure S1b**). Furthermore, the median size of the synapse as evidenced by Bsn staining was not altered in the synaptoneurosome preparation, suggesting that the protocol preserved synaptic structure, did not affect synaptic size and likely retained the structural diversity of the synaptic population (**Figure S1c**). In agreement with this, synapse-specific levels of Bsn were similar in the intact brain material and the synaptosomal preparation (**Figure S1d**). However, labelling for vGlut1 was drastically lower in the intact brain sample compared to the purified synaptosomal fraction, suggesting impaired penetration of the anti-vGlut1 antibody into the intact brain tissue (**Figure S1e**). Furthermore, coefficients of variation for vGlut1 and Bsn intensities in intact samples were considerable (72.36% and 55.85% respectively). Taken together, these data show that biochemically purified synaptoneurosomes enriched in the preparation are more amenable for immunostaining compared to intact brain samples, in agreement with previously published data^15,17^. Therefore, the above protocol was used for the rest of the study.

### Super-resolution microscopy

Samples were fixed and stained with Alexa Fluor 647 as for confocal imaging, except brain extracts were deposited in glass-bottomed chamber slides (#1.5 glass, ibidi μSlides) coated with PLL. For imaging, the final PBS wash was replaced with a volume of freshly prepared STORM imaging buffer (50 mM Tris-HCI (pH 8.5), 10 mM NaCl, 0.56M glucose, 5 U/ml pyranose oxidase (Sigma P4234), 40 μg/ml bovine catalase (Sigma C40), 35 mM cysteamine (Sigma 30070), and 2 mM cyclooctatetraene (Sigma 138924)). The dSTORM image sequences were acquired on a Nikon N-STORM 5 system in a TIRF configuration using a CFI SR HP Apochromat TIRF 100×AC 1.49 NA oil objective, arranged for a pixel size of 160 nm. Samples were illuminated with 647 nm laser light at approximately 2.05 kW/cm². Images were recorded using a Hamamatsu ORCA-Flash4.0 scientific CMOS camera using a centred 256 × 256 pixel region at 20 ms per frame for 15-20,000 frames.

### Super-resolution microscopy data analysis

Data were processed using ThunderSTORM^21^ version ‘dev-2015-10-03-b1’ and the following parameters: pre-detection wavelet filter (B-spline, scale 2, order 3), initial detection by non-maximum suppression (radius 1, threshold at one standard deviation of the F1 wavelet), and sub-pixel localization by integrated Gaussian point-spread function (PSF) and maximum likelihood estimator with a fitting radius of 3 pixels. Detected points were corrected for sample drift using cross-correlation of images from 5 bins at a magnification of 5. The occurrence of repeated localizations, such as can occur from long dye on times or fast re-blinking, was reduced by merging points (in the drift-corrected dataset) which reappeared within 50 nm and 25 frames of the initial detection. For the purposes of interpretation, it is assumed that the frequency of multiple detection of dye molecules is independent of the sample staining and therefore the relative changes in the clustering of points between sample conditions are independent of dye re-blinking. The merged dataset was then filtered with points retained according to the following criteria: an intensity range of 450 - 10000 photons, a sigma range of 50 - 250, and a localization uncertainty of less than 25 nm. The final dataset was then exported to a comma-delimited text file.

Data were analysed with CAML, a machine learning tool for SMLM cluster analysis recently developed by us^11^. Two SMLM images were manually annotated to label points within synapse-like structures. These data were then used to train a model to identify points which are both clustered and in synapse-like structures. This model, designated ‘2XOGG3’, demonstrated 98% accuracy in identifying points within synapses in the test dataset. The model was then used to annotate the remaining SMLM images and extract quantitative information on the clusters within synapses.

### Statistical analysis

All of the synapses automatically detected within these fields of view were included in the analysis. Statistical analysis was carried out using the Prism 6.0c software package (GraphPad Software). Data distributions were assessed for normality using d’Agostino and Pearson omnibus normality tests. All tests were unpaired and two-tailed. For normally distributed datasets, Student’s t-test, 1-way ANOVA and Holm-Šidák’s post-test were used to assess statistical significance as appropriate; for not normally distributed datasets, Mann-Whitney rank test, Kruskal-Wallis test and Dunn’s post-test were used for assessing statistical significance as appropriate. Datasets were presented as scatter dot plots with line at median or as cumulative probability plots, with error bars showing interquartile ranges where appropriate.

## Supplementary Figures

##

**Figure S1. Characterization of the synaptic preparation**. **a**, Schematics of the preparation of neurosynaptosomes. **b**, Colocalization between synaptic markers in neurosynaptosomes. **c**, Median synaptic area in neurosynaptosomal fraction (Fraction) and non-homogenised samples (Whole) from 4 brains. P=0.3881, t-test. **d**, Synaptic Bsn labelling in neurosynaptosomal fraction and non-homogenised samples from 4 brains. Intensities were normalised to neurosynaptosomal fraction. P=0.5036, one sample t-test. **e**, Synaptic vGlut1 labelling in neurosynaptosomal and non-homogenised samples from 4 brains. Intensities were normalised to neurosynaptosomal fraction. **P=0.0012, one sample t-test.

**Figure S2. Supporting data for Table 1.1**. **a**, Age is not significantly different between groups. P=0.7263 (male), P=0.5430 (female), P=0.7097 (both), 1-way ANOVA. **b**, PMI is not significantly different between groups P=0.8849 (male), 0.6154 (female), 0.7826 (both), 1-way ANOVA. **c**, Postsynaptic/presynaptic ratio does not correlate with PMI. P=0.4341, r=-0.1675, Spearman’s correlation coefficient.

**Figure S3. Supporting data for synaptic marker proteins levels 1. a**, Bsn synaptic levels in control, AD and DLB samples from male and female brains P=0.5210, 1-way ANOVA. **b**, median synaptic area in samples from control, AD and DLB male and female brains P=0.1005, 1-way ANOVA. **c**, RIM synaptic levels in sample from control, AD and DLB female and male brains P=0.3139, 1-way ANOVA. **d**, vGlut1 synaptic levels in samples from control, AD and DLB female and male brains, second sample preparation. **P<0.01, *P<0.05, 1-way ANOVA and Holm-Šidák’s post-test. **e**, Homer synaptic levels in sample from control, AD and DLB female and male brains. P=0.7449, 1-way ANOVA.

**Figure S4. Supporting data for Table 1.2**. **a**, Age of AD cases was significantly higher than that of control cases. *P<0.05, Student’s t test. **b**, 1-way ANOVA divided sex and condition shows no significant differences. P=0.1176, 1-way ANOVA. **c**, PMI is not significantly different. P=0.6761, t-test. **d**, R_A/D_ does not correlate with age of cases. P=0.1374 (Control), P=0.7429 (AD), r=-0.4526 (Control), r=0.1058 (AD), Spearman’s correlation coefficient.

**Figure S5. Supporting data for clustering experiments**. **a**, Schematics of the ratiometric clustering assay – adapted from Ref. 8. **b**, Area of Bsn clusters in control and AD samples; pairwise comparison is a two-tailed nested t test. **c**, Localization counts for Bsn in control and AD samples; pairwise comparison is a two-tailed nested t test.

**Table 1. Summary of clinical cases.**

**1.1. Main cohort.**

| **BBN ID** | **Sex** | **Age** | **PMI** | **Pathology diagnosis** | **AD Braak stage** | **ApoE genotype** | **Brain pH** |
| --- | --- | --- | --- | --- | --- | --- | --- |
| BBN_2925 | F | 86 | 14 | Alzheimer's disease (modified Braak VI) and amyloid angiopathy and limbic TDP43 pathology | VI | 3,4 | 6.56 |
| BBN_2926 | M | 82 | 69 | Alzheimer's disease limbic stage (modified Braak IV) with amyloid angiopathy and focal TDP43 pathology | IV | 3,4 | 7.14 |
| BBN_2932 | M | 80 | 10 | Alzheimer's disease Braak VI BNE 5 | VI | 4,4 | 6.33 |
| BBN_2936 | F | 84 | 24 | Alzheimer's disease BNE stage IV (Limbic stage AD) with focal amyloid angiopathy | IV | 3,3 | 6.74 |
| BBN_2937 | F | 85 | 55 | Alzheimer's disease Braak VI | VI | - | 6.31 |
| BBN_9900 | M | 88 | 5 | Alzheimer's disease Braak 6 with amyloid angiopathy | VI | 3,4 | 6.5 |
| BBN_9904 | M | 91 | 25 | Alzheimer’s disease Braak V, BNE IV; | V | 3,3 | 6.1 |
| BBN_9919 | F | 90 | 70 | Alzheimer's disease modified Braak 5;Focal TDP-43 pathology (predominantly limbic) | V | 3,3 | 5.98 |
| BBN_10280 | F | 82 | 43 | Pathology consistent with ageing | I | 3,3 | 6.33 |
| BBN_15616 | M | 86 | 6 | Ageing changes only | N/A | 3,3 | 6.6 |
| BBN_15752 | M | 79 | 24 | Mild ageing changes | N/A | 2,3 | 5.95 |
| BBN_16236 | F | 89 | 65 | Control case but with Hypoxic-type changes and amyloid angiopathy (AD BNE modified Braak score I-II) | I-II | 3,4 | 6.43 |
| BBN_16242 | F | 90 | 74 | control brain (modified Braak stage II) and mild amyloid angiopathy | II | 3,3 | 5.94 |
| BBN_16291 | M | 81 | 42 | control - old cerebral infarct (Braak I) | I | 3,3 | 6.72 |
| BBN_16579 | M | 85 | 16 | Ageing changes only | N/A | 2,2 | 6.61 |
| BBN_16629 | F | 80 | 31 | Mild ageing changes | N/A | 3,4 | 6.36 |
| BBN_2933 | F | 92 | 53 | Dementia with Lewy bodies (diffuse neocortical); mild limbic TDP-43 pathology. LB Braak stage 5. AD Braak 5 | V | 3,4 | 6.73 |
| BBN_10286 | M | 86 | 8 | Dementia with Lewy bodies (neocortical Lewy body disease). AD Braak 2 | II | 3,3 | 6.32 |
| BBN_15745 | M | 83 | 38 | Dementia with Lewy bodies (early Alzheimer’s changes) | III | - | - |
| BBN_16245 | F | 87 | 33 | Dementia with Lewy bodies (diffuse neocortical stage); Early Alzheimer’s disease changes (modified Braak score II) and focal amyloid angiopathy | II | 3,3 | 6.13 |
| BBN_16336 | F | 83 | 14 | Dementia with Lewy bodies (diffuse neocortical type); moderate Alzheimer's disease changes Braak IV | IV | - | 6.19 |
| BBN_16353 | M | 74 | 18 | Dementia with Lewy bodies (diffuse neocortical) | I-II | 3,3 | 6.71 |
| BBN_16362 | M | 88 | 17.5 | diffuse neocortical Lewy body dementia; Alzheimer's disease Braak V/VI | V/VI | - | - |
| BBN_16373 | F | 92 | 55 | Diffuse neocortical Lewy body disease; Argyrophilic Grain disease Braak III | III | - | 6.59 |

**1.2. Additional control and AD cases.**

| BBN_1889 | F | 85 | 19 | Alzheimer's disease Braak VI | VI | - | 5.9 |
| --- | --- | --- | --- | --- | --- | --- | --- |
| BBN_9918 | F | 88 | 22 | Alzheimer's disease modified Braak V with amyloid angiopathy | V | 3,4 | 5.95 |
| BBN_2934 | M | 97 | 16.5 | Alzheimer's disease Braak V, CERAD definite; TDP-43 pathology (predominantly in amygdala & hippocampus) | V | 3,4 | 6.18 |
| BBN_2935 | M | 88 | 12.5 | Alzheimer's disease Braak VI BNE V CERAD definite | VI | - | 6.45 |
| BBN_16536 | M | 80 | 11 | Minimal ageing changes | N/A | 3,3 | 5.88 |
| BBN_16522 | M | 77 | 29 | Mild ageing pathology | I-II | 3,3 | 6.71 |
| BBN_16601 | F | 79 | 38 | Pathology consistent with ageing | N/A | 3,3 | 6.7 |
| BBN_15609 | F | 68 | 9 | Very mild ageing changes | N/A | 3,3 | 6.02 |

**Table 2. Primary antibodies used in the study.**

| **Target** | **Species** | **Manufacturer** | **Cat. No.** | **Dilution** | **Validation** |
| --- | --- | --- | --- | --- | --- |
| Bassoon | Mouse | Abcam | 82958 | 1/1000 | ^22^ |
| Homer | Rabbit | Synaptic Systems | 160002 | 1/1000 | ^23^ |
| Gephyrin | Rabbit | Synaptic Systems | 147008 | 1/500 | ^24^ |
| vGlut1 | Rabbit | Synaptic Systems | 135302 | 1/2000 | ^25^ |
| RIM | Rabbit | Synaptic Systems | 140203 | 1/500 | ^26^ |
| Ca_V_2.1 | Rabbit | Synaptic Systems | 152103 | 1/500 | ^27^ |

## Supplementary references

16. Melvin NR, Sutherland RJ. Quantitative caveats of standard immunohistochemical procedures: Implications for optical dissector-based designs. *J Histochem Cytochem*. 2010;58(7):577-584. doi:10.1369/jhc.2009.954164

17. Hardy JA, Dodd PR, Oakley AE, Kidd AM, Perry RH, Edwardson JA. Use of post-mortem human synaptosomes for studies of metabolism and transmitter amino acid release. *Neurosci Lett*. 1982;33(3):317-322. doi:10.1016/0304-3940(82)90392-5

18. Jhou JF, Tai HC. The Study of Postmortem Human Synaptosomes for Understanding Alzheimer’s Disease and Other Neurological Disorders: A Review. *Neurol Ther*. 2017;6(Suppl 1):57-68. doi:10.1007/s40120-017-0070-z

19. Biesemann C, Grønborg M, Luquet E, et al. Proteomic screening of glutamatergic mouse brain synaptosomes isolated by fluorescence activated sorting. *EMBO J*. 2014;33(2):157-170. doi:10.1002/embj.201386120

20. Richter KN, Wildhagen H, Helm MS, Ußling JE, Schikorski T, Rizzoli SO. Comparative synaptosome imaging: a semi-quantitative method to obtain copy numbers for synaptic and neuronal proteins. *Sci Rep*. 2018;8(1):14838. doi:10.1038/s41598-018-33130-6

21. Ovesný M, Křížek P, Borkovec J, Švindrych Z, Hagen GM. ThunderSTORM: A comprehensive ImageJ plug-in for PALM and STORM data analysis and super-resolution imaging. *Bioinformatics*. 2014;30(16):2389-2390. doi:10.1093/bioinformatics/btu202

22. Annamneedi A, Caliskan G, Müller S, et al. Ablation of the presynaptic organizer Bassoon in excitatory neurons retards dentate gyrus maturation and enhances learning performance. *Brain Struct Funct*. 2018;223(7):3423-3445. doi:10.1007/s00429-018-1692-3

23. Synaptic Systems - Homer 1. Accessed July 21, 2022. https://sysy.com/product/160002

24. Synaptic Systems - Gephyrin. Accessed July 21, 2022. https://sysy.com/product/147008

25. Synaptic Systems - VGLUT 1. Accessed July 21, 2022. https://sysy.com/product/135302

26. Zarebidaki F, Camacho M, Brockmann MM, Trimbuch T, Herman MA, Rosenmund C. Disentangling the Roles of RIM and Munc13 in Synaptic Vesicle Localization and Neurotransmission. *J Neurosci Off J Soc Neurosci*. 2020;40(49):9372-9385. doi:10.1523/JNEUROSCI.1922-20.2020

27. Nishimune H, Numata T, Chen J, et al. Active Zone Protein Bassoon Co-Localizes with Presynaptic Calcium Channel, Modifies Channel Function, and Recovers from Aging Related Loss by Exercise. *PLoS ONE*. 2012;7(6):e38029. doi:10.1371/journal.pone.0038029
