# Supplementary figures and images for: Structural synaptic signatures of Alzheimer's disease and dementia with Lewy bodies in the male brain
Source: Neuropathol Appl Neurobiol. 2022 Oct 9;49(1):e12852. doi: 10.1111/nan.12852 (PMC10092423; doi:10.1111/nan.12852)

# Figure S1

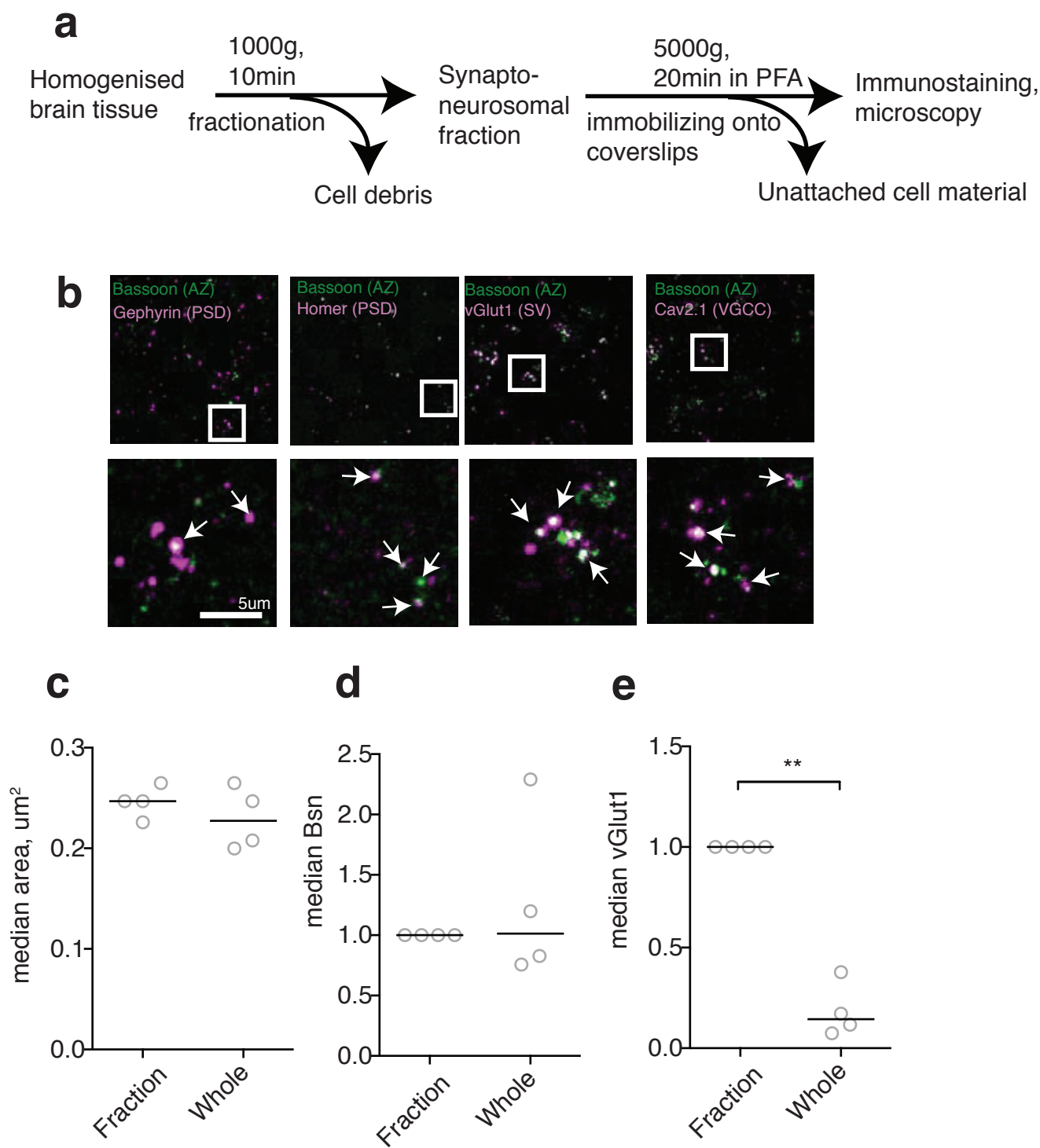

Supplement: Supplementary file 2 — Figure S1. Characterization of the synaptic preparation. a, Schematics of the preparation of neurosynaptosomes. b, Colocalization between synaptic markers in neurosynaptosomes. c, Median synaptic area in neurosynaptosomal fraction (Fraction) and non‐homogenised samples (Whole) from 4 brains. P = 0.3881, t‐test. d, Synaptic Bsn labelling in neurosynaptosomal fraction and non‐homogenised samples from 4 brains. Intensities were normalised to neurosynaptosomal fraction. P = 0.5036, one sample t‐test. e, Synaptic vGlut1 labelling in neurosynaptosomal and non‐homogenised samples from 4 brains. Intensities were normalised to neurosynaptosomal fraction. **P = 0.0012, one sample t‐test. [file NAN-49-0-s005.pdf]

**a**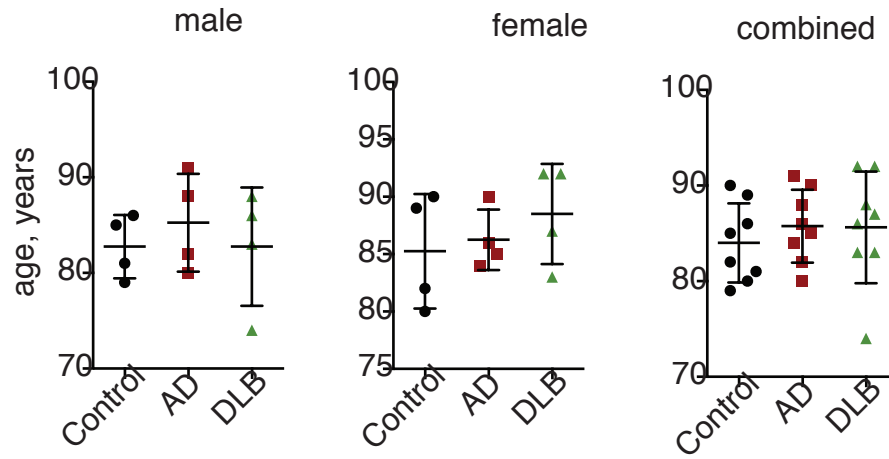**b**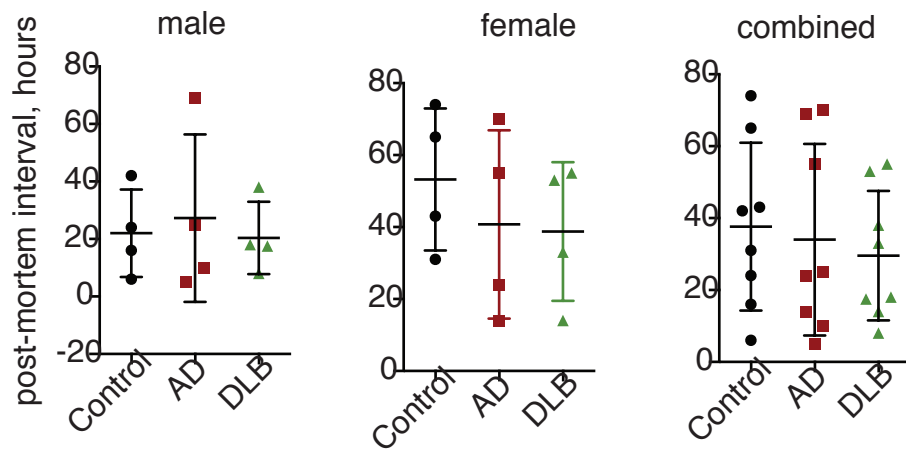**c**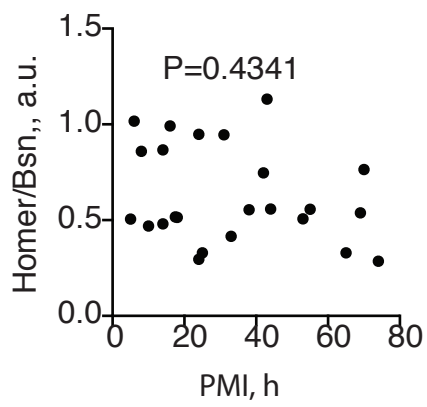

Supplement: Supplementary file 3 — Figure S2. Supporting data for Table 1.1. a, Age is not significantly different between groups. P = 0.7263 (male), P = 0.5430 (female), P = 0.7097 (both), 1‐way ANOVA. b, PMI is not significantly different between groups P = 0.8849 (male), 0.6154 (female), 0.7826 (both), 1‐way ANOVA. c, Postsynaptic/presynaptic ratio does not correlate with PMI. P = 0.4341, r = −0.1675, Spearman's correlation coefficient. [file NAN-49-0-s002.pdf]

**a**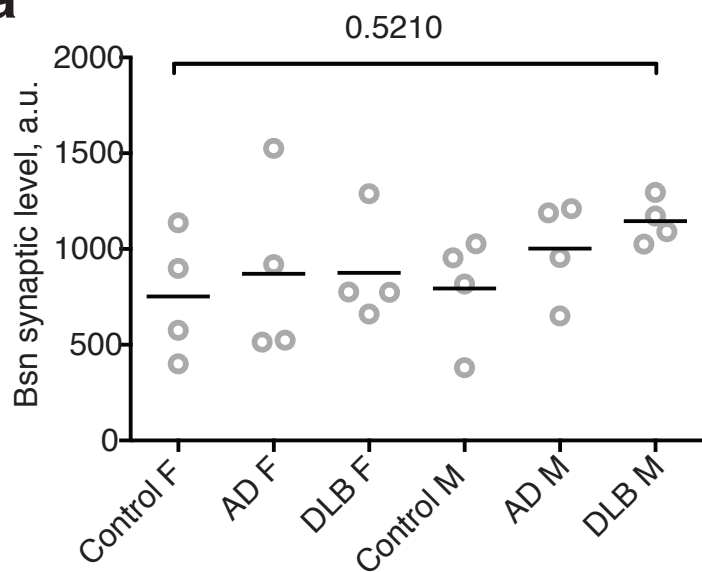**b**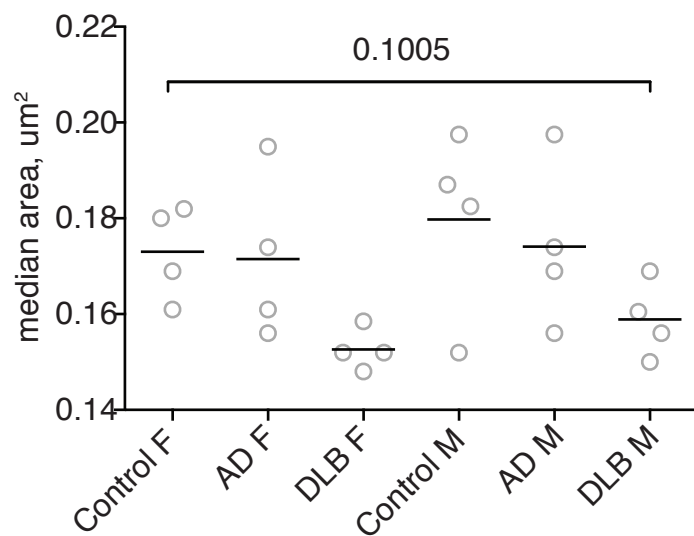**c**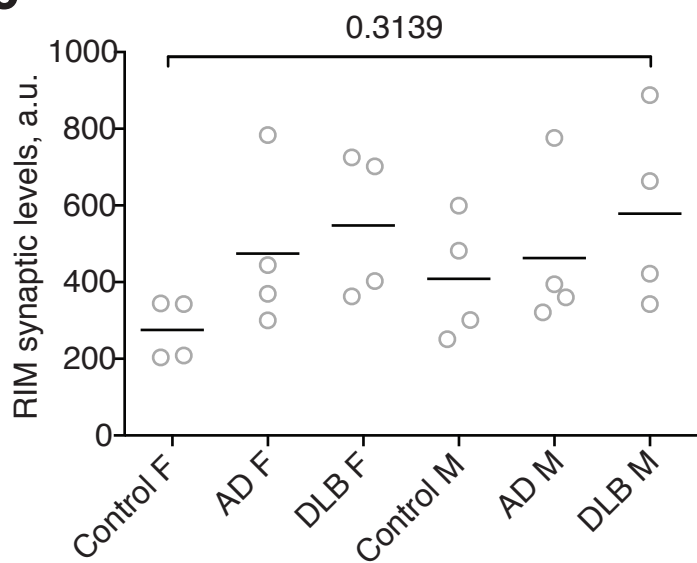**d**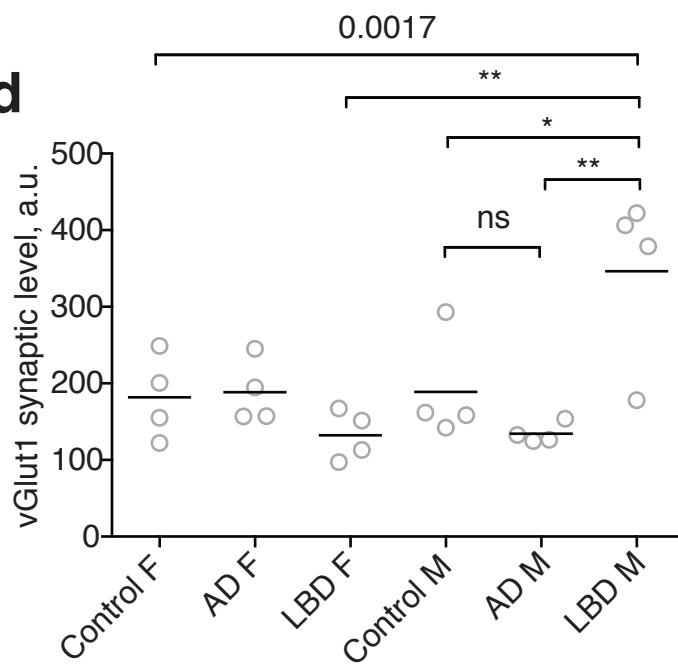**e**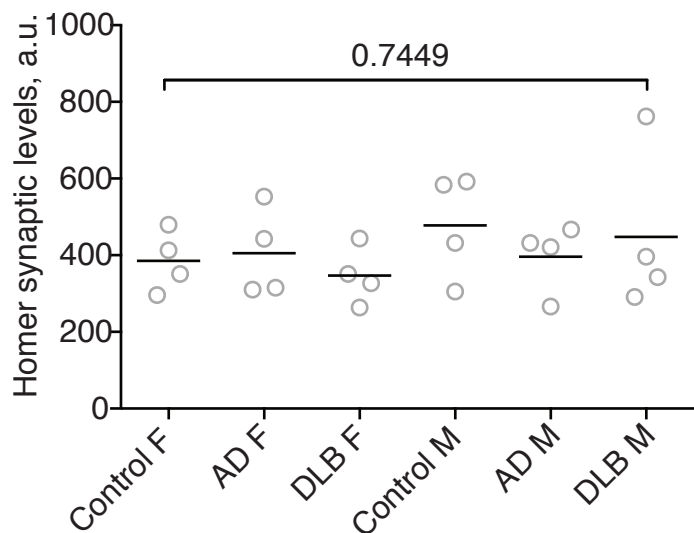

Supplement: Supplementary file 4 — Figure S3. Supporting data for synaptic marker proteins levels 1. a, Bsn synaptic levels in control, AD and DLB samples from male and female brains P = 0.5210, 1‐way ANOVA. b, median synaptic area in samples from control, AD and DLB male and female brains P = 0.1005, 1‐way ANOVA. c, RIM synaptic levels in sample from control, AD and DLB female and male brains P = 0.3139, 1‐way ANOVA. d, vGlut1 synaptic levels in samples from control, AD and DLB female and male brains, second sample preparation. **P < 0.01, *P < 0.05, 1‐way ANOVA and Holm‐Šidák's post‐test. e, Homer synaptic levels in sample from control, AD and DLB female and male brains. P = 0.7449, 1‐way ANOVA. [file NAN-49-0-s006.pdf]

Figure S4

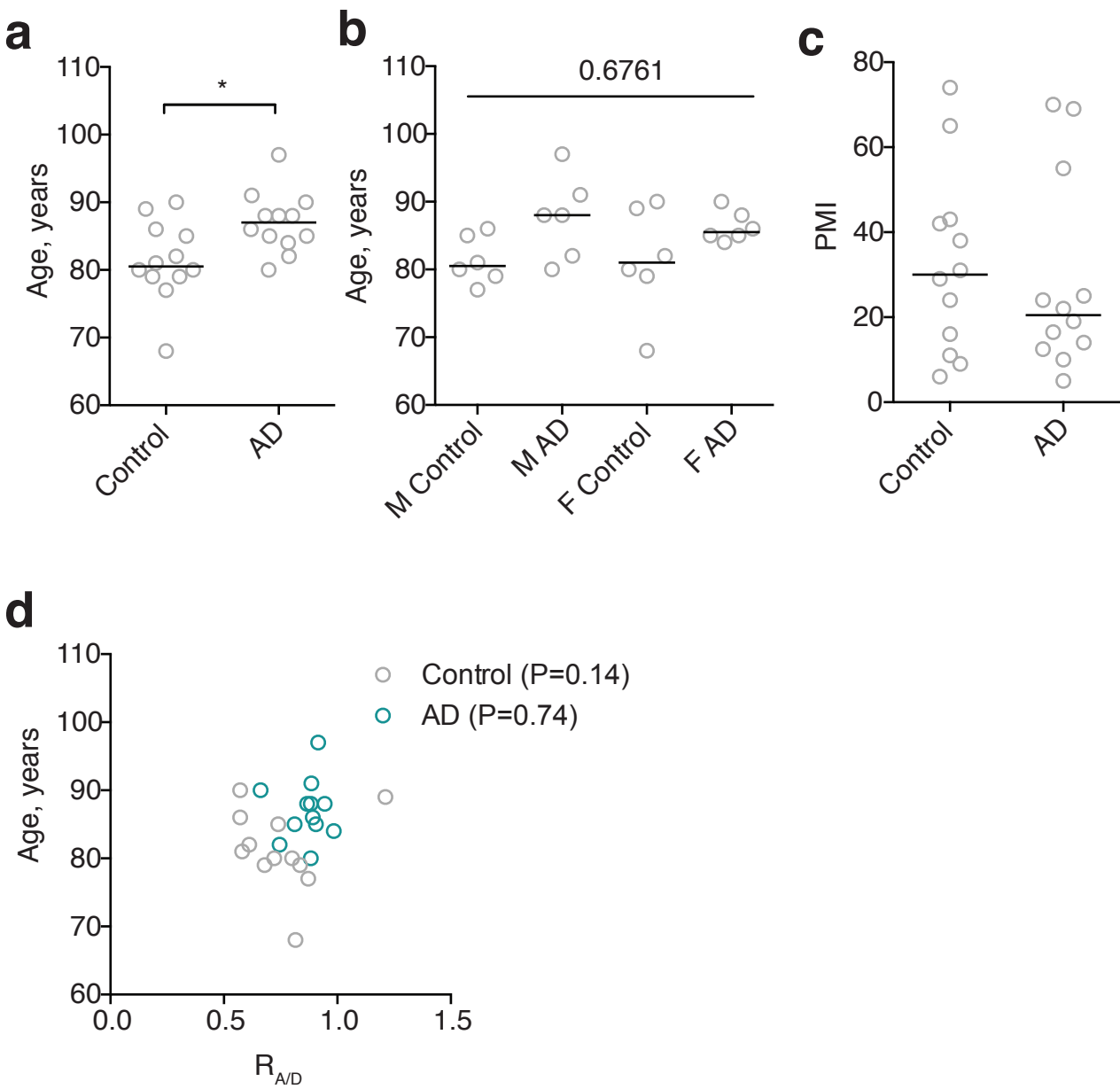

Supplement: Supplementary file 5 — Figure S4. Supporting data for Table 1.2. a, Age of AD cases was significantly higher than that of control cases. *P < 0.05, Student's t test. b, 1‐way ANOVA divided sex and condition shows no significant differences. P = 0.1176, 1‐way ANOVA. c, PMI is not significantly different. P = 0.6761, t‐test. d, RA/D does not correlate with age of cases. P = 0.1374 (Control), P = 0.7429 (AD), r = −0.4526 (Control), r = 0.1058 (AD), Spearman's correlation coefficient. [file NAN-49-0-s003.pdf]

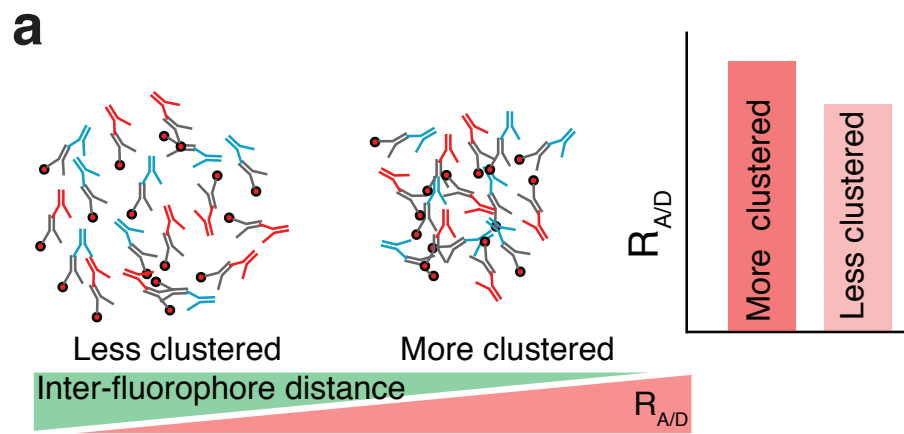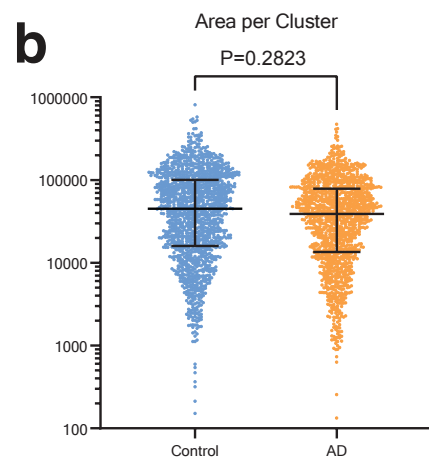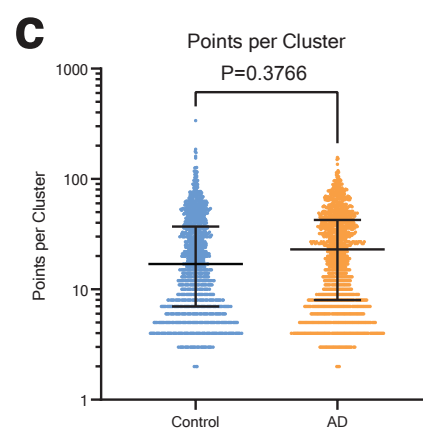

Supplement: Supplementary file 6 — Figure S5. Supporting data for clustering experiments. a, Schematics of the ratiometric clustering assay – adapted from Ref. 8. b, Area of Bsn clusters in control and AD samples; pairwise comparison is a two‐tailed nested t test. c, Localization counts for Bsn in control and AD samples; pairwise comparison is a two‐tailed nested t test. [file NAN-49-0-s001.pdf]
